# Supplementary material for: Identification of photoperiod-induced specific miRNAs in the adrenal glands of Sunite sheep (Ovis aries)
Source: Front Vet Sci. 2022 Jul 22;9:888207. doi: 10.3389/fvets.2022.888207 (PMC9354845; doi:10.3389/fvets.2022.888207)
Supplement: Supplementary file 1 [file Data_Sheet_1.ZIP › Supplementary file/miRDeep2 file.zip!/miRDeep2 file/Novel_Structure/SP42A3/Novel_168.pdf]

Provisional ID	: NC_019480.2_28892
Score total	: 1.8
Score for star read(s)	: 3.9
Score for read counts	: -4.4
Score for mfe	: 0.7
Score for randfold	: 1.6
Score for cons. seed	:
Total read count	: 3
Mature read count	: 2
Loop read count	: 0
Star read count	: 1

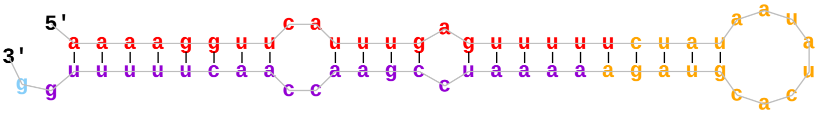

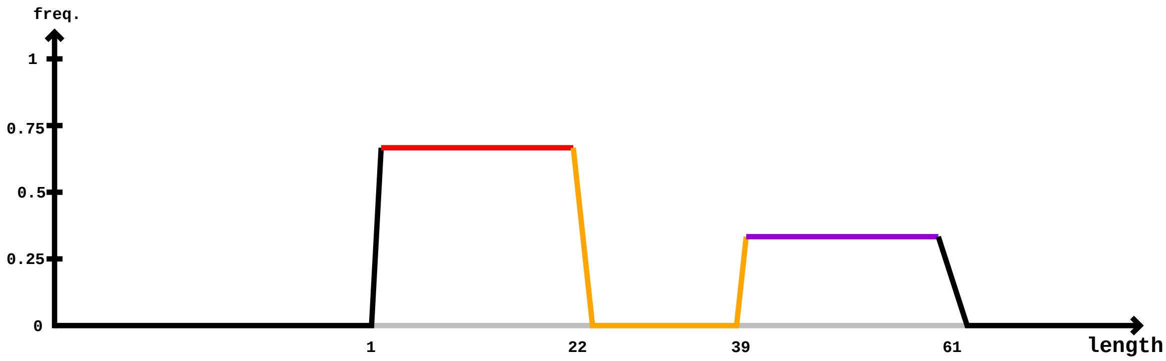

Mature Star

```
5'- uauuucuuccucaacauuauauuggguuagccaaaagguucauuugaguuuuucuauaauaucacguagaaaaauccgaaccaacuuuuuggucaagccaagaauugguau  -3'   obs
    uauuucuuccucaacauuauauuggguuagccaaaagguucauuugaguuuuucuauaauaucacguagaaaaauccgaaccaacuuuuuggucaagccaagaauugguau         exp
    ............((......((((.((.(((((((((((..((((.((((((((((........)))))))))).))))..))))))))))).)).))))......))..   reads   mm   sample
    ................................aaaagguucauuugGguuuuu..........................................................   2       1    seq
    ......................................................................aaaauccgaaccaacuuuuug....................   1       0    seq
```
